# Inter- and Intra-Species Communication of Emotion: Chemosignals as the Neglected Medium

**DOI:** 10.3390/ani9110887

**Published:** 2019-10-31

**Authors:** Gün R. Semin, Anna Scandurra, Paolo Baragli, Antonio Lanatà, Biagio D’Aniello

**Affiliations:** 1 Faculty of Social and Behavioral Sciences, Utrecht University, 3584 CS Utrecht, The Netherlands; gun.r.semin@gmail.com; 2 William James Center for Research, ISPA-Instituto Universitario, 1149-041 Lisbon, Portugal; 3 Department of Biology, University of Naples Federico II, Via Cintia, 80126 Naples, Italy; anna.scandurra@unina.it; 4 Department of Veterinary Sciences, University of Pisa, 56124 Pisa, Italy; paolo.baragli@unipi.it; 5 Department of Information Engineering, University of Pisa, 56122 Pisa, Italy; antonio.lanata@unipi.it

**Keywords:** communication, human, dog, horse, emotions, chemosignals

## Abstract

**Simple Summary:**

Human body odors contain chemosignals that make species-specific communication possible. Interspecies communication studies were conducted on dogs and horses subjected to human chemosignals produced in happiness and fear emotional states. Dogs showed behaviors consistent with human emotions, while horses exhibited differential activation of the autonomic nervous system. These results are leading the way for further studies on human–animal communication through emotional chemosignals.

**Abstract:**

Human body odors contain chemosignals that make species-specific communication possible. Such communication is without communicative intent and is generally below the threshold of consciousness. Human recipients of these chemosignals produced during emotional conditions display a simulacrum of the emotional state under which the chemosignal was produced. The investigation of an inter-species transfer of emotions via chemosignals was initiated by considerations of the historically anchored interdependence between humans and domesticated species, such as dogs and horses. Indeed, experiments with dogs have demonstrated that human body odors produced under emotional conditions of happiness and fear led dogs to manifest corresponding emotions to those experienced by humans. Preliminary data from horses also show that human body odors collected under fear and happiness conditions activate the autonomic nervous system of horses differentially. These studies indicate the possibility of a road to open our understanding of inter-species emotional communication via chemosignals.

## 1. Introduction

Communication is the most fundamental building block to all social species since it constitutes the core to social life as we know it - from humans [1,2] to honeybees (*Apis melifera*) [3,4,5,6] and from termites [7] to male stickleback fish [8] just to name a few examples. It is therefore not surprising that it has obtained considerable empirical attention. Diverse types of investigation have uncovered the extraordinary range of biological, neural, behavioral, and social factors that make communication possible and the wide range of shared media that enable species-specific communication, which could be acoustic, chemical, mechanical, optical, or some combination thereof. Although chemical communication is known to be widespread across the animal kingdom, the significant role it plays in humans has remained relatively neglected until very recently. Even less is known and researched when it comes to the contribution of chemosignals as evolutionarily conserved signals to interspecies transfer of information.

In this paper, we present an overview of the research highlighting the transfer of emotions via chemosignals amongst humans after a brief prelude on the beginnings of interest in human chemosignals. Next, we provide a summary of the emerging studies on the transfer of emotions from humans to domesticated animals via human chemosignals produced in emotion-inducing contexts. The final section will present some conclusions regarding the novel directions research might take in the field of interspecies communication.

### 1.1. Prelude: Human Chemosignaling

Across different disciplinary perspectives, the human olfactory sense has been regarded as the least significant of the senses [9]. Historically, the human capacity to communicate and sense via chemicals has been severely underestimated and misrepresented. This is partially due to the fact that unambiguous representations of odors (comparable e.g., to color terms) cannot be found in the vocabularies of most cultures [10] and recent research from Asida Majid and her colleagues [11] suggests that the distinct type of subsistence modes in specific Malay cultural communities has contributed to the emergence of odor vocabularies. The difference in the ease of olfactory naming is found to be related to cultural practices, namely the mobile hunter-gatherer Semaq Beri who compared to the more sedentary Semela shows considerable ease in odor naming. Inevitably, in the case of specific cultures (such as Western ones) the absence of a distinct and unambiguous linguistic representation for odors presents a major handicap in making odors objects of thought and linking olfactory sensations to (basic) linguistic representations.

Not having shared codes to categorize odors absents the representation of this sensory domain from any type of discourse. One consequence is to regard the human olfactory sense as being deficient compared to other senses. But this is at the core of underestimating the human capacity to discriminate between odors, which is remarkable. An earlier estimate [12] places the number of discriminable odorants at 400,000. More recently, this was estimated to be more than a trillion odors [13,14]. The recent estimates exceed people’s ability to discriminate colors [2.3–7.5 million] and tones [~340,000], though however, the higher estimated number is debated [15,16] and the actual number of discriminable olfactory stimuli is not known. Another source for the absence of an unambiguous odor coding is the question of how to estimate the dimensionality of odor space. It is not as easy to characterize and define the properties of odor space as in the case of sound (i.e., frequency, loudness) or color (i.e., wavelength, intensity). Odor space consists of a complex array [17] that defies easy classification.

The difficulties surrounding the representation of odor has not inhibited research on the diverse influences of human odors, revealing the communicative potential of human odors. There has been an increasing number of studies showing that humans can and do communicate with body odors. The type of information mediated by human odors range from emotional state [18,19,20,21], to illness [22], gender [23,24,25], sexual preference [26,27], sexual arousal [28], kin [29], and to mate compatibility [30].

### 1.2. Emotion Transfer from Humans to Humans via Chemosignals

Emotion contagion by visual and auditory media is well established, however a systematic approach towards elucidating human communication of emotional states via body odors (chemical signals) is of recent date. This research shows that human sweat not only communicates negative states (e.g., fear, disgust) [31], which arguably have putative adaptive functions but also positive emotions (i.e., happiness) [32]. The basic finding shows that a senders’ sweat produced in a specific emotional context induces a similar affective state in a receiver as measured by psychophysiological, cognitive and behavioral methods. Importantly, this mode of communication escapes conscious access, which has important implications for inducing affective states.

Research on the transmission of emotional information via body odors produced in different emotion-inducing contexts appeared a few decades ago (i.e., reference [18]). Generally, this research relies on odors collected from the armpit, while odor donors are exposed to films or activities inducing different emotional states (e.g., fear, disgust, and happiness) [31,32]. The measures of emotion transmission evidenced in recipients are often psychophysiological (e.g., electromyograph abbreviated as EMG from here on, testing the different facial muscles corresponding to different emotion expressions), behavioral tasks (e.g., reaction times, visual scanning), or response scales. Numerous studies examining the type of information carried by fear sweat have shown that the odor activates the medial frontalis muscle [20,23,31,32] or the corrugator supercilii muscle [20,23,33]. In the case of armpit odors of donors produced during a happy state, recipients have been shown to display activation of the orbicularis oculi, the Levator labii, and the Zygomaticus [32].

Aside from these types of differential facial muscle activation, other converging measures taken at the same time as EMG measures indicate the intricately convergent nature of the communicated message. One example is the type of cognitive processes activated by happy odors versus fear odors in recipients [32]. Thus, when exposed to odors induced during happy episodes, recipients process information more globally or abstractly (e.g., by using general concepts) when compared with exposure to fear odors where information is processed more locally or piecemeal (see reference [34] and references therein). Similarly, when recipients were exposed to disgust and fear odors, not only were EMG measures taken but also sniffing behavior, eye movements, and visual search were examined [31]. These indicators were introduced as measures of distinct patterns of facial behavior induced by fear which activate a sensory acquisition state [35] in contract to a disgust state, which induces a sensory rejection state. Thus, in the case of a fear state activated in a recipient, it was shown that the visual field was expanded, the visual search covered a broader area, and the predicted depth of inhalation behavior was found to be greater compared to during an induced disgust state in recipients [31]. Moreover, Kamiloğlu, et al. [36] showed that a fear odor induces a discrete fear state in its recipient rather than a general negative evaluative state. Participants were exposed to facial images of anger, disgust, fear, as well as neutral expressions, which gradually changed from completely noisy images to fully clear images. They were able to identify fear images much earlier than all the other images when they were exposed to a fear odor rather than a neutral odor. These results are indicative of categorical processing when exposed to fear odor, rather than the odor activating a general negative evaluative process, as the latter would have suggested a general processing advantage for all negative images.

### 1.3. Emotion Transfer from Humans to Other Animals

The general argument about the function of body odors is that they constitute chemical signals that evolved for species-specific communication [37,38,39]. Notably, chemoreception is the oldest sense that preceded the evolution of specialized sensory systems (e.g., vision, audition) and is universal. It is, therefore, possible to argue that chemosignals in general and emotion chemosignals, in particular, may not only be a species-specific medium for communication but also play a role in interspecies communication. In other words, one species’ signal may not merely be a cue for another species but may also act as a signal. Indeed, it has been suggested that the sense of smell occupies a special place in locating our evolutionary past, as the brain regions dedicated to odor processing constitute the oldest structures in mammalian evolution [40].

Synanthropic species, such as dogs and horses, are particularly good at dealing with interspecies chemosignaling. This came from a long domestication process in close proximity with humans, allowing them to trust humans and cooperate, learn, and communicate with them [41,42]. The onset of domestication for dogs is dated at about 19,000 years ago, as showed by molecular data [43]; whereas the earliest evidence of horse domestication traces back to about 6000 years ago [44]. Although horses and dogs arise from progenitors with a very different behavioral ecology, namely prey and predators, they have converged in acquiring similar skills in communicating with humans.

Due to evolutionary plasticity, brain changes can take place very quickly [45,46], leading to the evolution of cognitive processes that optimized the adaptation of dogs to human society in a relatively short period. This evolutionary adaptation was the outcome of the co-evolution of dogs with humans which was fostered by their artificial selection. The consequence was that dogs evolved to treat humans almost as conspecifics [47]. Regardless of training and life stories, dogs can form a very strong attachment bond with their human companions [48,49,50,51], whereas there is limited support for the horse–human attachment [42]. In a communicative context, dogs developed a high sensitivity to human gestures [52,53,54], being able to also learn human words [55,56,57]. Notably, they can perceive and respond to human emotions through visual and auditory signals [58,59,60,61]. Similar skills have been achieved by horses. They are able to integrate different sensory systems to individually recognize both conspecifics and humans in a cross-modal recognition [62,63,64] and to combine different facial cues of conspecifics to gather information on the environment [65]. Moreover, horses can also communicate their emotions [66] and understand the facial expressions of both horses [67] and humans [68]. Some recent findings suggest that physiological measurements (e.g., heart rate variability) taken from humans and horses show a synchronization of these indicators as a function of the type of contact they had [69].

Siniscalchi and his colleagues have conducted specific investigations revealing that domesticated dogs have olfactory features that are specialized for specific human properties related to their visual expressions of emotion, as well as their odors secreted under specific emotions such as anxiety. When exposed to human faces expressing anger, fear, and happiness emotions, dogs receive the visual stimuli of emotional expressions by turning their heads towards the left (right hemisphere) rather than the right, suggesting that the canine brain processes basic human emotions asymmetrically [70]. These findings converge with their earlier research [71] on canine nostril use when exposed to human odors produced under anxiety conditions. Their research with domestic male dogs recorded a clear bias in left or right nostril use when the dogs were inhaling human or canine anxiety odors, respectively. The dogs used opposite nostril sides when inhaling conspecific or human odors. Conspecific anxiety odors were accessed by the use of the right nostril, while human anxiety odors were sniffed with the left nostril.

The interesting question that can be raised in this context is: what are the behavioral effects that dogs exposed to human odors display when the odors are produced under emotion-inducing conditions such as fear or happiness? A recent study investigated precisely this, namely, if human odors produced in specific emotion-inducing contexts (i.e., happiness, fear) induce a simulacrum in a species with a long domestication history. This recent study by D’Aniello and colleagues [72], examined the interspecies transfer of emotions, namely from humans to dogs. Forty retrievers (Labrador and Golden) were exposed to human fear or human happiness odors in an experimental room (4 m by 3 m) with a water bowl in a corner and two chairs in the diagonal corners of the room (one for the owner and the other for a researcher, who was unknown to dogs). The dogs were individually exposed to the odors (happy, fear, control) through a protected vial in the center of the room. Their heartbeat rate and behaviors were monitored. The video recordings of the dogs were analyzed by two independent coders who were blind to the conditions. The general coding categories were approach, interaction, and gazing behaviors that were directed towards the apparatus, the owner or the stranger. Additionally, data were obtained via a heart rate monitor that was strapped around the chest of the dogs. The discriminant analysis with the obtained variables yielded two discriminant functions that yielded a high degree of accuracy (87.5%) with the a priori classifications of the dogs to the odor conditions (happy, fear, control) suggesting that the behaviors and heart beat rate (HBR) were distinctly different as a function of the odor conditions. A happy human odor evoked ‘happy behaviors’ from the dogs (e.g., approach and interaction behaviors with stranger), and human fear odor triggered behaviors indicative of dog fear states (e.g., approach and interaction behaviors with owner), possibly activating the attachment system. Notably, the precise interpretation of the mechanism driving these effects remains unclear. These findings indicate that the emotional information transfer process between different species may be due to different processes. One possible mechanism may be that volatility has a distinct chemical composition that activates emotion specific behaviors and physiological states. The alternative mechanism that can account for the same outcomes may be the result of an associative learning process, whereby the dogs are able to identify a distinct odor that is emitted with a distinctive behavioral syndrome. Notably, the odors in question (i.e., happiness, fear) in this study were collected in Portugal from a sample of males between the ages of 18 and 35. The dogs were in Italy, Napoli, where the reported research was conducted. If the findings are due to an associative learning process, then this would attest to a remarkable olfactory learning capacity of dogs. However, the learning process alone cannot explain the results reported by D’Aniello et al. [72], as the human odors were collected from Portuguese adults aged between 18 and 35. In D’Aniello et al.’s [72] case, different dogs reared and living in Italy were exposed to fear or happiness odors of different humans living in Lisbon, Portugal. This means the systematic effects obtained in this study cannot be explained by familiarity with odors - which are shaped by diets that are different from those that the dogs would be used to. In other words, the human chemosignal has an invariant property and the ‘situated meaning’ of this invariant is acquired by the dogs. These findings therefore suggest that there is likely a distinctive chemical fingerprint in the volatiles that is particular to the emotional context in which it is produced. This however does not mean that the distinctive chemical fingerprint to fear and happiness induces an innate response. It is very likely that the dogs are sensitive to the specific odor and learn to ‘recognize’ the emotion associated with the specific volatile.

More recently, Lanatà et al. [73] were able to show a similar contagion effect with a small sample of horses who were exposed to happiness and fear odors. These highly tentative results converge with the research reported by D’Aniello et al. [72]. In this study, human body odors were shown to induce systematic sympathetic and parasympathetic changes showing that the body odors carry the emotional information to horses. The data that they obtained reveal that the human chemosignals induced a similar emotional status in horses. However, although these findings could open the way to understanding the behavior of horses when interacting with humans [74,75], they remain highly speculative at this stage and invite replication with an adequate sample size.

## 2. Discussion and Conclusions

The intraspecies and interspecies transfer of emotional state studies we reviewed raises a number of questions deriving from the assumption that chemosignals may constitute evolutionarily conserved signals. An important one is what drives emotional transfer between and within species? One of the two candidate answers is that the odors produced in emotional contexts (of fear and happiness) contain distinctive chemical compounds that invariably activate the same responses across the two species and thus have a pheromone type quality. The second possible answer is that the responses to the odor are acquired in the process of socialization and the two species acquire a sensitivity to the specific emotion-induced odors. As we argued earlier on, if this second process is responsible, it is nevertheless the case that there is a very distinctive emotion quality to the odor compound to the extent that in the study examining the transfer of emotional states from humans to dogs the volatiles were—as we noted—collected in Portugal and administrated to the pet dogs in Italy. Moreover, recent research [76] has revealed that emotional transfer extends beyond ethnocultural boundaries. In their study, East Asian (and Western Caucasian) female receivers were exposed to body odors collected from Western Caucasian male senders in fearful, happy, and neutral contexts. The facial expressions of the samples from the two groups of receivers revealed the same systematic muscle activities displayed under the three emotion conditions.

Collectively, these studies suggest that there are distinctive chemical fingerprints, independent of the senders’ and the receivers’ cultural community. Moreover, the same behavioral and physiological effects are found across humans and pet dogs. These systematic findings suggest that there is chemical invariance in the sweat compounds. What remains open is if such invariant compounds activate an automatic emotional response or whether the emotional responses are learned.

The studies showing interspecies transfer raise further questions. One of these is whether the interspecies emotion transfer is unidirectional as in the case of the findings reported by D’Aniello et al. [72] (i.e., from human to other animals) or if it is bidirectional (humans to other animals reciprocally). Testing the ‘dog and horses to human’ transfer of emotion would involve collecting animal body odors from the body and salivary secretions under different emotion conditions (e.g., fear) and exposing human participants to the animal odors to compare the outcome with findings from the human to another animal study. This approach would permit an examination of the bidirectionality of chemosignal aided transfer of information, at least in the emotion area. Obviously, the triangulation of this process would be very important. This would be achieved by examining the possibility of a chemosignal driven transfer of emotional information from interspecies communication.

A further set of questions related to the generality of such chemosignal driven transfer beyond humans to other species. We have some tentative evidence, as we mentioned with another domesticated example of species, namely horses. To examine the possibility of evolutionarily conserved signals, it would be important to examine the effects of emotion carrying human odors on not domesticated progenitors, such as wolves.

This type of cross-species research allows conceptualizing communication from a broader perspective signaled by the emerging research on chemosignals as the medium of communication, with the notable observation that we know the medium but do not the notation that carries this mode of communication. The extraordinary significance of olfactory communication discussed here relies on the argument that these constitute evolutionarily conserved signals driving biological sensory systems critical for survival and reproductive success. Their special power is to be found in their significance in promoting survival chances, especially where visual and acoustic senses are restricted. It is in the absence of these sensory functions that odors become uniquely powerful by providing silent and invisible warning signals with long-lasting effects.
